# Effect of Hyposalivation on Fish Cake Mastication in Healthy Individuals

**DOI:** 10.1002/fsn3.71378

**Published:** 2026-01-19

**Authors:** Chisato Aizawa, Reiko Ita, Yuto Ochiai, Anna Sasa, Namon Phetnin, Yoko Kawana, Kazuhiro Ono, Natsuka Takada, Takanori Tsujimura, Jin Magara, Makoto Inoue

**Affiliations:** ^1^ Division of Dysphagia Rehabilitation Niigata University Graduate School of Medical and Dental Sciences Niigata Japan; ^2^ Department of Speech, Language, and Hearing Sciences Niigata University of Health and Welfare Niigata Japan; ^3^ Division of Oral Science for Health Promotion, Department of Oral Health and Welfare Niigata University Graduate School of Medical and Dental Sciences Niigata Japan; ^4^ Technical Research Department Ichimasa Kamaboko Co., Ltd. Niigata Japan; ^5^ Unit of Dysphagia Rehabilitation Niigata University Medical & Dental Hospital Niigata Japan

**Keywords:** atropine, bolus property, electromyography, fish cake, hyposalivation, mastication

## Abstract

During mastication, solid food is mixed with saliva to form a bolus. However, little is known about the effects of hyposalivation on mastication. The effects of hyposalivation on masticatory behavior were investigated using fish paste products. Healthy volunteers (*n* = 21) were instructed to eat two fish paste products (fish cakes and rolled omelets) in a natural manner. These foods are elastic with high water content. Electromyographic activity was recorded in the masseter, suprahyoid, and infrahyoid muscles during mastication before and after atropine administration, which reduced salivary flow. Masticatory dynamics, including electromyographic activity and masticatory cycle time, were compared between volunteers with and without hyposalivation, and among the early, middle, and late masticatory stages. Next, the volunteers (*n* = 19) were instructed to ingest the food in the same manner and then spit it out at the end of mastication. After atropine administration, spitting was repeated, and bolus properties, such as hardness, water content, and piece size, were compared between the conditions. Hyposalivation did not affect any fish cake sensory evaluation properties; however, masticatory duration for rolled omelet increased considerably. Furthermore, no significant differences were observed between volunteers with and without hyposalivation in terms of masticatory cycle time, muscle activity per cycle at each stage, bolus physical properties, and bolus water content. Owing to the high water content of fish paste products, such as fish cake or rolled omelet, hyposalivation may not substantially affect mastication of these products.

## Graphical abstract

Hyposalivation did not affect any fish cake sensory evaluation properties. Masticatory duration for rolled omelet increased considerably. No significant differences in fish cake were observed between volunteers with and without hyposalivation, owing to the high water content of fish paste products.

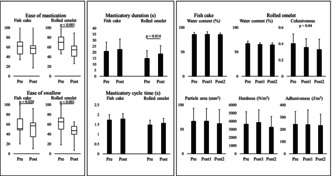

## 1 Introduction

Mastication is the first step of digestion. During mastication, food is reduced in size, saliva is secreted to moisten the food, and flavors are released. Moreover, salivary mucins bind small food particles into a coherent mass that can be safely swallowed.

The initiation of swallowing depends on separate thresholds for food particle size and lubrication (Hutchings and Lillford 1988; Prinz and Lucas 1997). Peyron et al. (2002) showed that bolus hardness rapidly decreased with time during mastication, whereas adhesiveness, springiness, and cohesiveness regularly increased until the first swallow, partly due to mixing with saliva. The authors suggested that physical changes occurred, with sensory stickiness as the dominant perception of the bolus at the end of mastication. Thus, salivation may be an important functional factor in both mastication and the ready‐to‐swallow stage. This may be critical, especially for older adults whose salivation is affected by a variety of factors, such as aging itself or diseases (Vandenberghe‐Descamps et al. 2017). Therefore, the association between reduced salivary function and food consumption should be considered in this population (Muñoz‐González et al. 2018). Furthermore, dry mouth is strongly associated with quality of life, thus strengthening the value of monitoring dry mouth conditions in the care of frail older adults (Gerdin et al. 2005).

Not only physical properties, such as hardness, but also salivation or food water content influence mastication. Dry and hard products require more masticatory cycles before swallowing because more time is needed to break down the food and mix saliva into a bolus suitable for swallowing (Anderson et al. 1985). Sasa et al. (2022) suggested that the water absorption rate changes suprahyoid muscle activity and mandibular movements, especially during the late stage of mastication. Hence, the higher the water absorption rate of the bolus, the more complex the masticatory movement patterns. During mastication of rice crackers, masticatory behaviors, including masticatory duration until the first swallow, number of masticatory cycles, masticatory cycle duration, and suprahyoid muscle activity, were markedly influenced by atropine‐induced hyposalivation (Goto et al. 2024). Furthermore, fat content compensates for the effects of hyposalivation on mastication. Other food characteristics, such as the fat percentage of solid foods, may also influence this process (van der Bilt and Abbink 2017).

Fish paste products, such as kamaboko, are traditional Japanese foods consisting of various types of Japanese fish cakes. These cakes are prepared using fresh fish meat or processed white fish called “surimi” as the main ingredient. In the production process, fish are crushed, minced, and mixed with flavors and starchy ingredients, as well as other food materials, before being fried. Rolled omelets, named datemaki in Japan, are a type of traditional fish cake made from fish paste and eggs. Rolled omelets are typically served as a Japanese New Year celebration food.

The characteristic features of fish cake, especially kamaboko, are its chewy, elastic, and firm texture. It can be assumed that even after mastication, small pieces of kamaboko particles remain in the oral cavity. Although most Japanese older adults enjoy these traditional foods, they sometimes avoid them or are discouraged from eating them because of their age, masticatory functional decline, and general health. Oral health encompasses daily abilities, such as mastication, swallowing, respiration, speaking, and expressing emotions (Glick et al. 2016). Therefore, poor oral health with decreased masticatory function can have detrimental effects on overall health and mortality (Tanaka et al. 2018).

In this study, the possible effects of hyposalivation on fish cake mastication were investigated based on the functional role of saliva. This study aimed to investigate how a reduction in salivary flow affects mastication in healthy young volunteers. The study hypothesized that, because of the unique physical property of fish cake, impaired salivation does not affect masticatory function.

## 2 Materials and Methods

In this study, two experiments were conducted: electromyography (EMG) and spitting tests. Prior to the experiment, a dentist conducted examinations of all participants, confirming no clinical difficulties with eating behaviors and no missing teeth except for the third molars. Written informed consent was obtained from all participants and approval was obtained from the Ethics Committee of Niigata University (approval no. 2020‐0039). All experiments were performed according to the Ethical Principles of the Declaration of Helsinki for Medical Research Involving Human Participants (2024).

### 2.1 EMG Test

#### 2.1.1 Participants

A total of 21 healthy adults, including twelve males and nine females, ranging from 24 to 54 years (average age ± standard deviation [SD], 28.6 ± 3.8 years), participated in the study. The inclusion criteria were as follows: no history of any structural disorders or medical diseases, such as alimentary, pulmonary, or neurological diseases.

#### 2.1.2 Test Foods

Two commercial fish cakes were prepared as test foods. Commercially available fish paste products included fish cakes, named kamaboko in Japan (Ichimasa Kamaboko Co. Ltd., Niigata, Japan) and rolled omelets, named datemaki in Japan (Ichimasa Kamaboko Co. Ltd.). The characteristics of the foods are listed in Table 1. Water content was measured using a moisture analyzer (ML‐50, A&D Company Ltd., Tokyo, Japan) according to a previous study (Takei et al. 2020).

**TABLE 1:** Characteristics of the foods.

	Fish cake	Rolled omelet
Energy (g/100 g)	86.3	212.0
Protein (g/100 g)	8.4	9.2
Fat (g/100 g)	0.3	6.5
Carbohydrates (g/100 g)	12.8	29.1
Sodium chloride equivalent (g/100 g)	1.6	0.9
Water content (%)	78.42	53.76

Compression tests were conducted using a food rheometer (RE2–33005S, Yamaden, Tokyo, Japan) (Kohyama et al. 2004). Briefly, a cylindrical specimen (22 × 12 × 4 mm) was compressed between a cylindrical probe (diameter = 8 mm) and a stage at a constant rate of 1 mm/s and a strain of 97% of the initial height. The sample fracture was determined at the point at which the first stress reduction was observed. The sample hardness was expressed as the fracture stress, calculated as the detected load divided by the initial contact area of the probe and sample (30.8 mm^2^ for cylindrical probes). The relative deformation was calculated as the ratio of the penetration depth to the initial height of each sample. Fracture energy was calculated as the energy required to break the sample. This test was repeated more than five times for each food item. The data were analyzed using the Creep Analysis software package (version 2.0; Yamaden, Tokyo, Japan).

#### 2.1.3 Data Acquisition

The recording procedure is described in a previous study (Goto et al. 2024). Briefly, electrodes (ZB‐150, Nihon Kohden Co. Ltd., Tokyo, Japan) to record surface EMG activity were attached to the following surfaces: the skin over the masseter and anterior belly of the digastric muscle for the left and right suprahyoid muscles, and the thyrohyoid muscle for the left infrahyoid muscles. EMG signals were amplified and filtered; high‐pass and low‐pass filter cutoff frequencies were 30 Hz to remove movement‐related artifacts and 2 kHz, respectively (Web‐1000 EMG Medical Telemeter; Nihon Kohden). The EMG signals were stored on an interface board (PowerLab; AD Instruments, Colorado Springs, CO, USA) on a computer (1 kHz). Data were analyzed using the PowerLab software package (LabChart 8; AD Instruments, Colorado Springs, CO, USA).

Prior to the experiment, the participants were instructed to not eat or drink for at least 1 h. The subjects were seated upright without a headrest support throughout the recording period. First, reference EMG data for each muscle was obtained. Each participant was instructed to bite three times with maximal force for 3 s and to maximally open the jaw three times for 3 s. An interval of at least 10 s was provided between tasks.

Next, the first masticatory task in which participants were asked to freely ingest either of the two foods (7.0 g) in random order was conducted, and EMG activity was recorded. The interval between trials was set at 2 min, and the participants were allowed to rinse their mouths with water whenever they wanted. After the first task, atropine sulfate (1 mg; FUJIFILM Wako Pure Chemical Corporation, Osaka, Japan) was orally administered to induce hyposalivation. The atropine sulfate dose was determined based on a previous study (Goto et al. 2024). Unstimulated saliva was collected immediately before and every 10 min for 60 min after atropine administration. The weight of the unstimulated secreted saliva was measured using the cotton roll test as previously described (Goto et al. 2024). Briefly, a cotton roll was placed at the base of the tongue for 30 s. During this period, participants were instructed to not move their jaws and tongues as much as possible. The cotton roll was removed from the oral cavity and weighed. A second masticatory task was conducted in the same manner 40 min after atropine administration. Because the masticatory task took more than 10 min, salivary flow 50 min after atropine administration was not recorded. Finally, EMG activity was recorded again during 3 s of maximum biting and 3 s of maximum jaw opening 60 min after atropine administration.

#### 2.1.4 Sensory Evaluation of Mastication and Swallowing

The ease of mastication and swallowing with and without hyposalivation was compared. The participants were asked to rate the ease of mastication and swallowing using a visual analog scale, with scores ranging from 0 (difficult mastication/swallowing) to 100 (easy mastication/swallowing).

#### 2.1.5 Data Analysis

Physical properties of samples such as fracture stress (MPa), relative deformation at fracture (%) and fracture energy (kJ/m^3^) were compared using a *t*‐test.

The salivary flow rate was compared between the time points using Friedman repeated‐measures analysis of variance by rank, followed by Dunnett's test for further analysis. For the analysis of sensory evaluation, the ease of mastication and swallowing was compared before and after atropine administration using the Wilcoxon signed‐rank test.

For EMG analysis, all waveforms were full‐wave rectified and smoothed (time constant, 20 ms). The mean value ± SD of EMG activities at rest for 5 s was obtained. EMG burst was considered active when it exceeded the mean + two SDs. EMG activity was defined as the area under the curve of the filtered EMGs. To confirm the reproducibility and reliability of the EMG data, the EMG activity and power frequency of the EMG bursts of the masseter and suprahyoid muscles were compared between 1st and 2nd reference tasks using intraclass correlation coefficients (ICC).

Masticatory duration (s) was defined as the time between the onset of the first masticatory cycle and the offset of the masticatory cycle immediately before the first swallowing, as previously described (Sasa et al. 2022; Goto et al. 2024). The onset of one masticatory cycle was defined as the onset of the right masseter EMG burst. A pharyngeal swallowing event was identified as an infrahyoid EMG burst and visually confirmed by laryngeal elevation. For further analysis, the mean masticatory duration until first swallow and mean masticatory cycle time with and without hyposalivation were compared for each food sample using a paired *t*‐test.

Next, the temporal changes in masticatory behavior were evaluated to clarify how EMG activity changes with time as previously described (Goto et al. 2024; Takei et al. 2020). Each masticatory sequence, up to the first swallow in each trial, was proportionally divided into three stages depending on the number of masticatory cycles. Three cycles at the early (from the second to fourth masticatory cycles), middle (the median three cycles during mastication), and late stages (the last three masticatory cycles before swallowing) were selected. Masticatory cycle time and EMG activity per masticatory cycle at each stage were compared using a two‐way repeated‐measures analysis of variance (ANOVA) or Friedman two‐way repeated‐measures ANOVA on ranks (stage × hyposalivation), followed by Tukey's honestly significant difference (HSD) test. In this procedure, the EMG amplitudes of the masseter and suprahyoid muscles were normalized to the mean value of the EMG activity obtained from the 1st biting and jaw‐opening tasks, respectively. Further, normalized masseter and suprahyoid EMG data from the left and right sides were averaged.

The sample size was calculated using G*Power 3.1, indicating that at least 18 healthy participants with complete datasets were required to achieve a statistical power of 95% and a *p*‐value of < 0.05. Statistical analysis was performed using SigmaPlot software (SigmaPlot 15.0, Systat Software Inc., San Jose, CA, USA) and Bell Curve for Excel (Social Survey Research Information Co. Ltd., Tokyo, Japan). Statistical significance was set at *p* < 0.05. All values are expressed as mean ± SD except the masticatory cycle time and EMG activity (mean ± standard error of the mean).

### 2.2 Spitting Test

#### 2.2.1 Participants

This experiment was performed on a separate day but at the same time as the EMG test. A total of 19 healthy adults, including nine males and ten females, ranging from 24 to 38 years (average age ± SD, 30.4 ± 4.2 years) participated. The inclusion criteria were as follows: no history of any medical diseases, such as alimentary, pulmonary, or neurological diseases, or structural disorders.

#### 2.2.2 Data Acquisition and Analysis

First, participants were asked to freely ingest either of the two foods (first masticatory task) in random order, and the masticatory duration until the first swallowing was recorded (first masticatory duration). They ingested the food again in the same manner, followed by spitting it out at the end of mastication, which was determined by the first masticatory duration at the cue of the examiner (first spitting task). Ten minutes later, the weight of the unstimulated secreted saliva was measured using a cotton roll test. Subsequently, atropine sulfate (1 mg) was administered orally. Unstimulated saliva was collected every 10 min for 60 min after atropine administration, except at 50 min. A second masticatory task was conducted in the same manner 40 min after atropine administration. After unstimulated saliva was collected 40 min after atropine administration, the second masticatory duration was recorded, followed by a spitting task. Considering that the masticatory duration was conducted twice, before and after atropine administration, spitting tests were also performed twice at these time points (second and third spitting tasks) for each food sample based on the first and second masticatory durations after atropine administration. The time interval between the trials was at least 2 min, and the subjects were able to rinse their mouths with distilled water whenever they wished.

Similar to the EMG test, the salivary flow rate was compared between the time points using Friedman's repeated‐measures analysis of variance by rank, followed by Dunnett's test for further analysis. Masticatory duration with (second masticatory task) and without hyposalivation (first masticatory task) was compared between participants using a paired *t*‐test.

Given that fish cakes have unique elastic properties, they are not completely minced and mixed with saliva during mastication. Instead, fish cakes are torn into small pieces; hence, 10 pieces were randomly selected and the area was measured using ImageJ (Schneider et al. 2012). The water content of each bolus was measured using a moisture analyzer (ML‐50, A&D Instruments, Tokyo, Japan) (Takei et al. 2020). The rheological bolus properties of the rolled omelets after mastication were measured using a creep meter (RE2‐33005S; Yamaden, Tokyo, Japan) with a modified two‐bite test (Hino et al. 2024). Samples were placed on the plate (40 mm diameter, 15 mm height) and elevated toward a plunger (20 mm diameter, 8 mm height) at a speed of 10 mm/s. The plunger was connected to a load cell that depressed the sample twice (compressibility 66.7%). Thereafter, we calculated the values for hardness (the height of the first peak in the stress–strain curve), adhesiveness (the area of the first negative peak, i.e., the work required to pull the plunger away from the food sample), and cohesiveness (the ratio of the area under the second compressive peak to the area under the first compressive peak). Data were analyzed using Creep Analysis software package version 2.0 (Yamaden Co. Ltd., Tokyo, Japan). For fish cakes, particle area and water content were compared among the conditions obtained from the first, second, and third spitting tasks using a one‐way repeated‐measures ANOVA, followed by Tukey's HSD test. For rolled omelets, water content and bolus properties were compared among the conditions in the same way.

Statistical analysis was performed using SigmaPlot software (SigmaPlot 15.0; Systat Software Inc., San Jose, CA, USA) and BellCurve for Excel (Social Survey Research Information Co. Ltd., Tokyo, Japan). A *p* value < 0.05 was considered significant. All values are expressed as the mean ± standard deviation or median (IQR 25%–75%), except those for the masticatory cycle time and EMG activity per masticatory cycle (mean ± standard error of the mean).

## 3 Results

### 3.1 Physical Properties of Food Samples

The data are summarized in Table 2. The fracture stress of the fish cake was larger than that of the rolled omelet, whereas the relative deformation at fracture of the fish cake was smaller than that of the rolled omelet. Overall, the fracture energy was higher for fish cake than for rolled omelet.

**TABLE 2:** Physical properties of the food samples.

	Fish cake	Rolled omelet
Fracture stress (MPa)	0.36 ± 0.01	0.23 ± 0.01***
Relative deformation at fracture (%)	88.72 ± 1.05	96.13 ± 0.08***
Fracture energy (kJ/m^3^)	83.6 ± 4.5	39.4 ± 2.2***

*** *p* < 0.001.

### 3.2 Change in Salivary Flow

**FIGURE 1:**
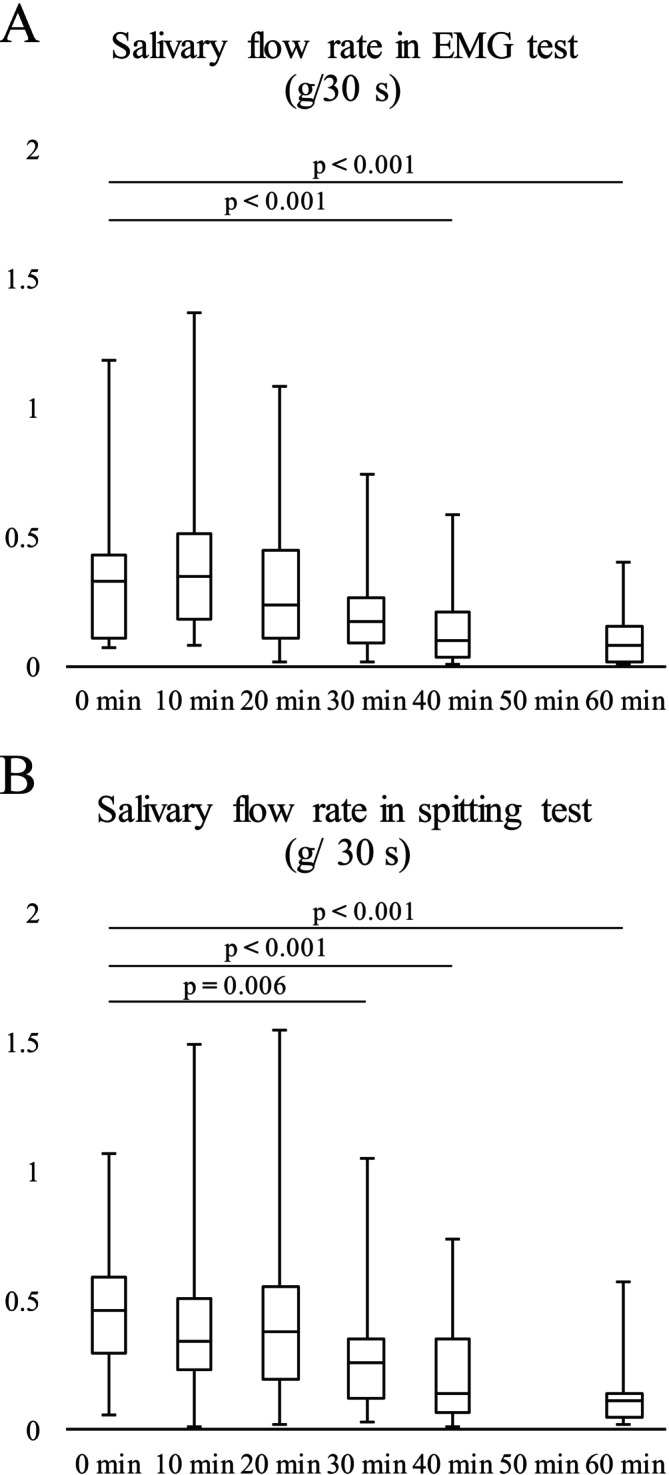
Change in the salivary flow rate. Salivary flow rate was recorded separately in electromyography test (A) and spitting test (B). (A) The median (interquartile range [IQR]: 25%–75%) salivary flow rate (g/30 s) was 0.33 (0.11–0.43) at 0 min (before atropine administration), 0.34 (0.19–0.52) at 10 min, 0.24 (0.11–0.45) at 20 min, 0.18 (0.09–0.27) at 30 min, 0.11 (0.03–0.21) at 40 min, and 0.09 (0.02–0.16) at 60 min. (B) The median (IQR: 25%–75%) salivary flow rate (g/30 s) was 0.46 (0.29–0.59) at 0 min (before atropine administration), 0.34 (0.23–0.50) at 10 min, 0.37 (0.19–0.55) at 20 min, 0.26 (0.12–0.35) at 30 min, 0.14 (0.06–0.35) at 40 min, and 0.10 (0.04–0.14) at 60 min. Data were analyzed using Friedman's repeated‐measures analysis of variance by rank, followed by Dunnett's test (*n* = 19).

Salivary flow gradually decreased over time. In the EMG test, significant differences were observed between 0 and 40 min (*p* < 0.001) and between 0 and 60 min (*p* < 0.001) (Figure 1A). This was also the case for the spitting test, in which there were significant differences between 0 and 30 min (*p* = 0.006), 0 and 40 min (*p* < 0.001), and 0 and 60 min (*p* < 0.001) (Figure 1B). The participants were monitored for more than 2 h after atropine administration, and none reported any discomfort.

### 3.3 Ease of Mastication and Swallowing Sensory Evaluation

**FIGURE 2:**
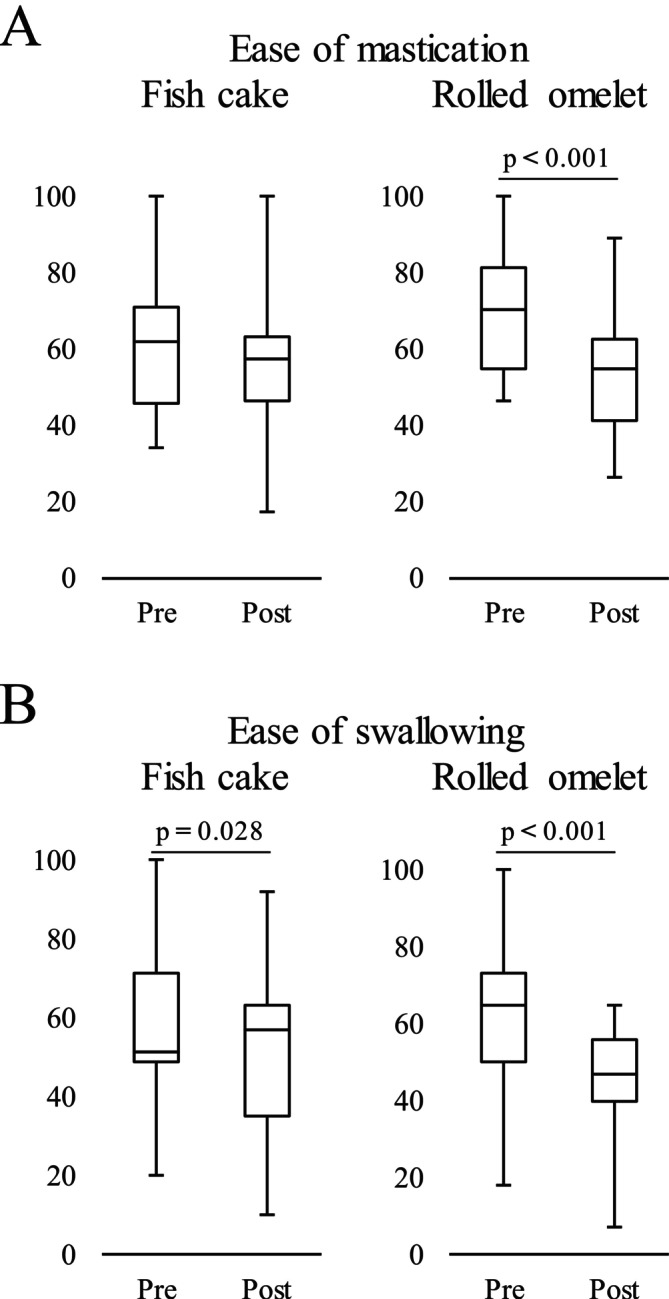
Effect of hyposalivation on ease of mastication and swallowing. (A) The median (interquartile range [IQR]: 25%–75%) ease of mastication score was 62% (45.75%–70.75%) without hyposalivation and 57.5% (46.5%–63%) with hyposalivation for fish cake, and 70% (54.75%–81%) without hyposalivation and 54.5% (41.25%–62.5%) with hyposalivation for rolled omelet. (B) The median (IQR: 25%–75%) ease of swallowing score was 51% (48.5%–71.25%) without hyposalivation and 57% (34.75%–63%) with hyposalivation for fish cake, and 65% (50%–73%) without hyposalivation and 47% (39.75%–55.75%) with hyposalivation for rolled omelet. The data were analyzed using Wilcoxon signed‐rank test (*n* = 21). Pre, without hyposalivation; Post, with hyposalivation.

After atropine administration, ease of mastication significantly decreased only for rolled omelet (*p* < 0.001) (Figure 2A), whereas ease of swallowing significantly decreased for both foods (*p* = 0.028 for fish cake and *p* < 0.001 for rolled omelet) (Figure 2B).

### 3.4 Effect of Hyposalivation on Masticatory Behavior

To confirm the reproducibility and reliability of the EMG data, the EMG activity and power frequency of the EMG bursts of the masseter and suprahyoid muscles were compared between the first and second biting (for masseter muscles) and opening tasks (for suprahyoid muscle) using ICC as described above. The ICC were 0.882 (*F* = 15.90, *p* < 0.001) for the left masseter, 0.851 (*F* = 12.42, *p* < 0.001) for the right masseter, 0.840 (*F* = 11.47, *p* < 0.001) for the left suprahyoid, and 0.829 (*F* = 10.67, *p* < 0.001) for the right suprahyoid. The ICC of the power frequency of the EMG bursts were 0.701 (*F* = 5.69, *p* < 0.001) for the left masseter muscle, 0.670 (*F* = 5.06, *p* = 0.0011) for the right masseter muscle, 0.758 (*F* = 7.26, *p* < 0.001) for the left suprahyoid muscle, and 0.685 (*F* = 5,34, *p* < 0.001) for the right suprahyoid muscle. These results indicate excellent reliability; therefore, we concluded that no muscle fatigue occurred during recording.

**FIGURE 3:**
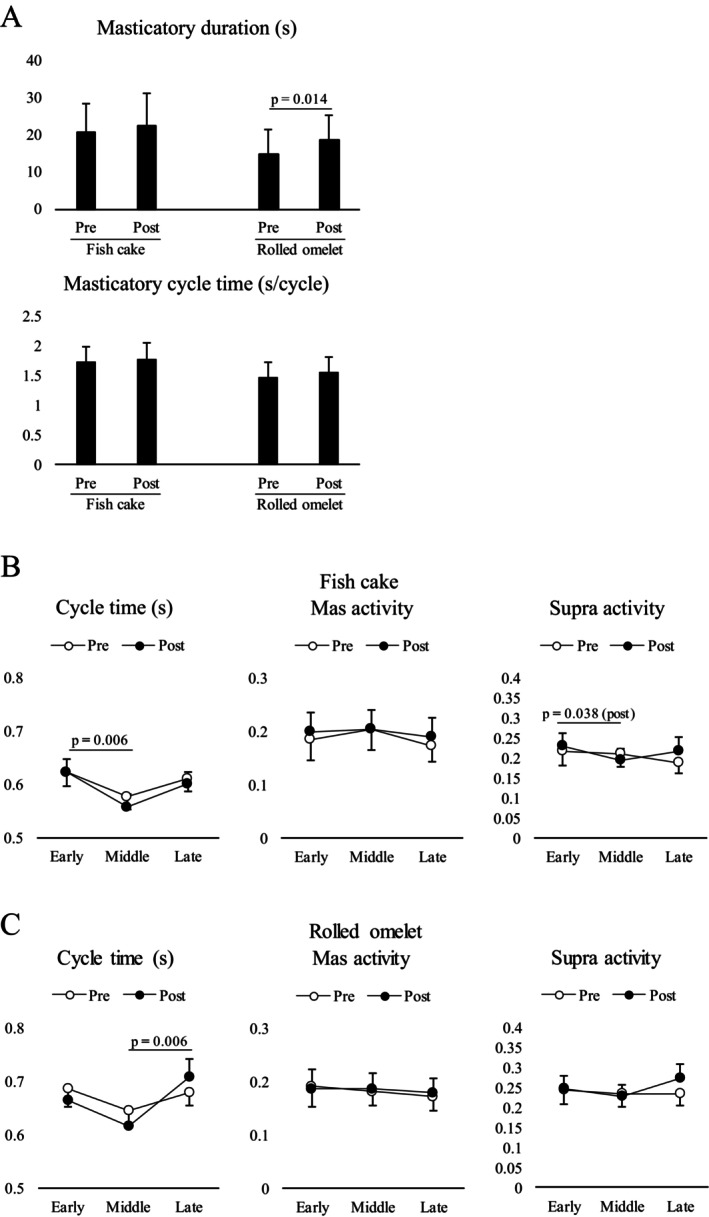
Effect of hyposalivation on masticatory activity. (A) The effect of hyposalivation on masticatory duration was observed only in rolled omelets. The data were analyzed using a paired *t*‐test (*n* = 19). Pre, without hyposalivation; Post, with hyposalivation. Temporal changes in masticatory cycle time and masseter and suprahyoid electromyography (EMG) activity per masticatory cycle for fish cake (B) and rolled omlet (C) were compared. No significant effect of hyposalivation on all the parameters was observed. The open and closed circles indicate before (pre) and after (post) atropine administration, respectively. The data were analyzed using two‐way repeated measures analysis of variance (ANOVA), followed by Tukey's honestly significant difference (HSD) test (*n* = 19).

The EMG data of two subjects was excluded from the analysis because their complete EMG signals were not recorded. In terms of fish cake mastication, the paired *t*‐test revealed no significant differences in masticatory duration or cycle time (Figure 3A). For rolled omelet, paired *t*‐tests revealed a significant difference in masticatory duration (*p* = 0.014), whereas no significant difference in masticatory cycle time was observed (Figure 3A).

### 3.5 Temporal Changes in Masticatory Behavior

Masticatory behavior may change over time in a masticatory sequence. Temporal changes in the masticatory cycle time and masseter and suprahyoid EMG activity per masticatory cycle before and after atropine administration were compared for each food item. Generally, the effect of hyposalivation on temporal changes was not observed in either the fish cakes or rolled omelets (Figure 3B,C).

For masticatory cycle time, two‐way repeated‐measures ANOVA with stage × hyposalivation revealed a significant effect of stage (F2, 38 = 5.692, *p* = 0.007) on fish cake and stage (F2, 38 = 5.657, *p* = 0.007) on rolled omelet. Further post hoc testing showed that the masticatory cycle time was significantly longer in the early stage than in the middle stage (*p* = 0.006) for fish cake and significantly longer at the late stage than at the middle stage (*p* = 0.006) for rolled omelet. For masseter activity per masticatory cycle, two‐way repeated‐measures ANOVA with stage × hyposalivation did not reveal a significant effect of stage or hyposalivation. In terms of suprahyoid activity, hyposalivation did not have a significant effect on stage or hyposalivation, with a significant interaction (F2, 38 = 3.279, *p* = 0.038) for fish cake only. Further post hoc testing showed that suprahyoid activity with hyposalivation was significantly higher at the early stage than at the middle stage (*p* = 0.038) for fish cake.

### 3.6 Correlation Between Changes of Masticatory Cycle Duration and Suprahyoid Activity

Notably, although no significant differences were observed between the masticatory behaviors with and without hyposalivation, the change in masticatory cycle time was likely affected by suprahyoid activity from the middle to late stages. Therefore, the correlation between changes in the masticatory cycle duration and suprahyoid activity from the middle to late stages was compared using Pearson product–moment correlations for the foods. A strongly positive relationship was observed between changes in suprahyoid activity and masticatory cycle time in all cases (Table S1). Correlation coefficient (CC) for both foods was significantly higher with hyposalivation (Post) than without hyposalivation (Pre) (*Z* = 2.708 and *p* = 0.007 for fish cake; *Z* = 2.119 and *p* = 0.034 for rolled omelets). The CC without hyposalivation was significantly larger for fish cakes than for rolled omelets (*Z* = 2.708 and *p* = 0.007), whereas the CC with hyposalivation was significantly larger for rolled omelets than for fish cakes (*Z* = 3.000 and *p* = 0.003).

### 3.7 Spitting Test

**FIGURE 4:**
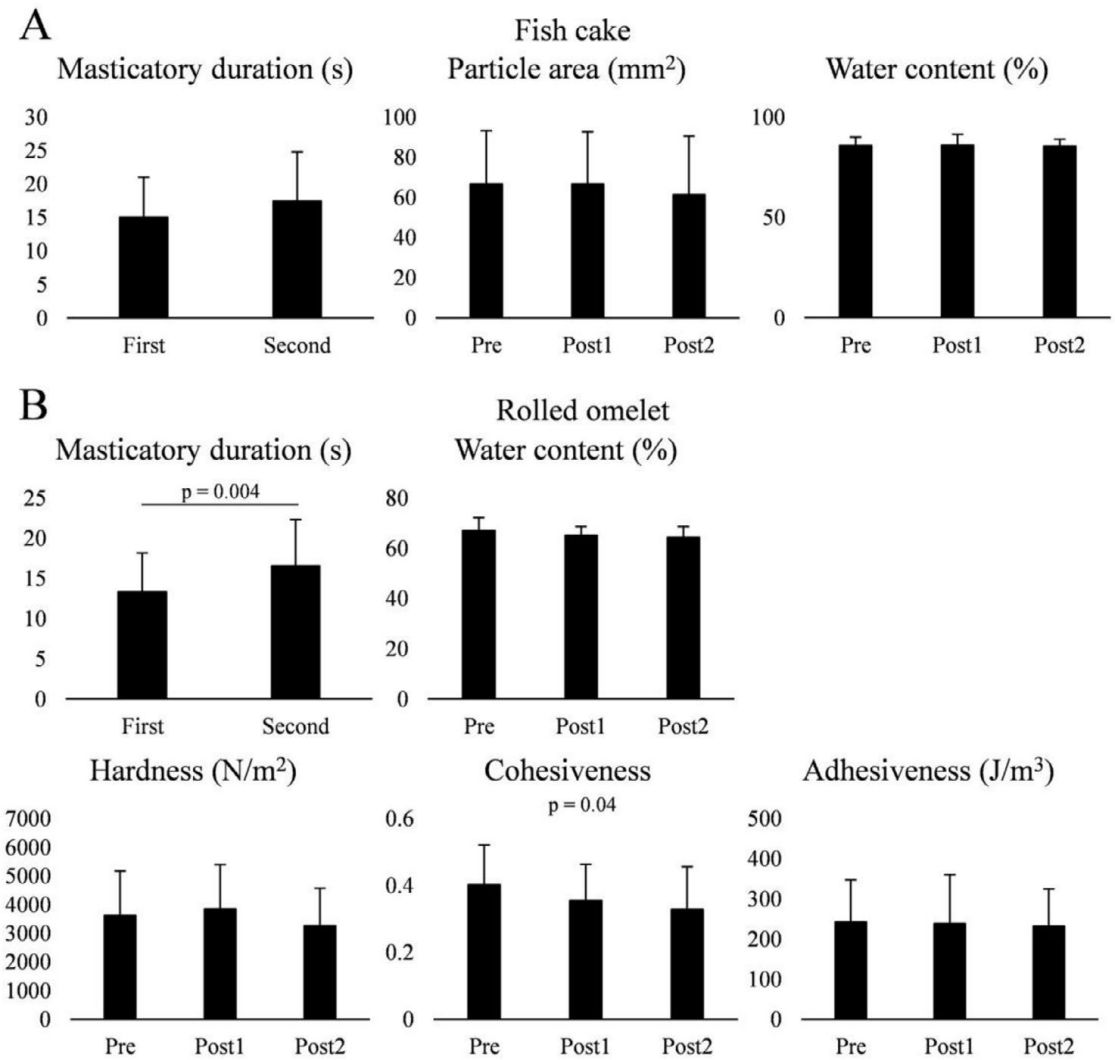
Effect of hyposalivation on bolus properties. (A) For fish cakes, no significant difference in the masticatory duration between the first and second mastication tasks was observed (*n* = 19, paired t‐test). In addition, the particle size and water content of the bolus did not differ among the conditions (*n* = 19, one‐way repeated‐measures analysis of variance [ANOVA]). (B) For rolled omelets, a significant difference in the masticatory duration was observed between the first and second mastication tasks (*n* = 19, paired *t*‐test). No significant difference in the water content was observed among the conditions (*n* = 19, one‐way repeated‐measures ANOVA). Regarding rheology, a significant difference in the cohesiveness was observed among the conditions (*p* = 0.04, *n* = 19, one‐way repeated‐measures ANOVA). Pre, before atropine administration with the masticatory duration derived from the first mastication task; Post1, after atropine administration with the masticatory duration derived from the first mastication task; Post2, after atropine administration with the masticatory duration derived from the second mastication task.

For fish cakes, no significant difference in the masticatory duration was observed between the first and second masticatory durations. In addition, no significant differences in the particle area and water content were observed among the three spitting tests (Figure 4A). In the case of rolled omelets, the first masticatory duration was significantly longer than the second masticatory duration (*p* = 0.004), which was consistent with the EMG test results (Figure 4B). Furthermore, no significant difference in water content was observed among the conditions. In terms of physical properties, although cohesiveness was considerably different among the conditions, further post hoc testing showed no significance.

## 4 Discussion

### 4.1 Effect of Hyposalivation on Masticatory Behaviors

Hyposalivation did not affect the mastication of fish cake in terms of masticatory duration or masticatory cycle time. However, the masticatory duration of rolled omelets increased considerably after atropine administration.

Fish cakes are consumed globally and most commonly consumed in Japan. Fish cake is rich in high quality protein and calcium and can be easily consumed without cooking. Therefore, even for older adults who have lost their skeletal muscle mass, fish cakes are a beneficial source of protein and are recommended to maintain general health. However, few studies have investigated the masticatory muscle activity involved in the mastication and swallowing of fish cakes (Ohguri et al. 1999; Fueki et al. 2013; Narita et al. 2015). These studies have investigated biomechanical properties during mastication; however, the effects of hyposalivation on fish cake mastication remain unclear.

Fish cakes exhibit a variety of textures depending on the manufacturing process. Food samples used in this study were characterized by their elastic textures. The fish cake was harder than the rolled omelet, whereas the latter was slightly more elastic than the former. Rolled omelets were completely crushed after mastication, forming a whole food bolus, whereas the fish cake remained in small pieces. Therefore, the bolus properties of these foods were analyzed using different methods.

Salivation is an essential process for bolus manipulation (Prinz and Lucas 1997; Peyron et al. 2017). The effect of impaired salivation on mastication depends on the food sample (Goto et al. 2024; Dusek et al. 1996; Liedberg and Owall 1991; Ochiai et al. 2025). Liedberg and Owall (1991) demonstrated that hyposalivation did not affect masticatory performance during the mastication of chewing gum or silicon, which are non‐swallowing materials, and markedly increased the number of masticatory cycles with almonds before swallowing. Goto et al. (2024) showed that after atropine administration to reduce salivary flow, the masticatory duration and number of masticatory cycles substantially increased in most rice crackers with low water content. Ochiai et al. (2025) demonstrated that while hyposalivation directly affected mastication behavior for dry bread, with increased masticatory duration, cycle time, and suprahyoid muscle activity, this was not the case for non‐dry bread. The authors concluded that bolus formation and transport during mastication of dry bread were interrupted by hyposalivation. Kohyama et al. (2005) demonstrated that masticatory behavior is substantially affected by the water content of food. They showed that the number of masticatory cycles, masticatory duration until the first swallow, and jaw‐closing muscle activities per masticatory cycle were the highest in cooked rice with the lowest water content of all cooked samples, whereas the masticatory cycle time did not differ. Although the differences in the initial properties of the foods should also be considered, water content may compensate for reduced salivary secretion.

Water absorption may also be an important factor in determining moisture content in the oral cavity. Takei et al. (2020) demonstrated that changes in masticatory behavior were affected by water absorption during bolus formation using rice crackers. This was expected because rice crackers are very dry, and water absorption directly affects the wet condition of the oral cavity. In this study, the water content did not change over time under both normal and dry conditions of the oral cavity. This strongly suggests that because neither food used in this study absorbed saliva, the water content did not change over time.

### 4.2 Temporal Change of Muscle Activity

The masticatory cycle time decreased slightly in the middle stage for both foods and was not affected by hyposalivation. Temporal changes in masticatory movements have previously demonstrated decreased masticatory cycle time in the middle stage, followed by an increase in the late stage, resulting in U‐shaped curves (Goto et al. 2024; van der Bilt and Abbink 2017; Takei et al. 2020). This suggests that careful manipulation is required to place food between the molars at an early stage. At this stage, complicated oral processing may be required, resulting in an increased masticatory cycle time. Furthermore, bolus formation and propulsion are needed, accompanied by an increase in the masticatory cycle time (Goto et al. 2024). This change in masticatory movements may be mainly due to lubrication rather than physical properties of the bolus. Suprahyoid activity increased with an increase in the masticatory cycle time at the late stage (Goto et al. 2024; Takei et al. 2020; Ochiai et al. 2025). However, in this study, no significant difference in suprahyoid activity was observed at the late stage and no effect of hyposalivation on suprahyoid activity was observed although the correlation between changes in the masticatory cycle duration and suprahyoid activity from the middle to late stages was observed for both fish cakes and rolled omelets. Similarly, the water content was quite high for both foods, and they did not absorb saliva during mastication. In terms of temporal changes in the masticatory cycle time, masseter activity per cycle, and suprahyoid activity per cycle, no effect of hyposalivation was observed. Considering the impact of bolus water content, it can be assumed that mastication was not substantially altered, even under hyposalivation conditions.

Masticatory behaviors were not considerably affected by hyposalivation, whereas masticatory duration markedly increased with hyposalivation for rolled omelets only. This difference is likely related to ease of mastication although the data were not precisely analyzed. In addition, the subjects complained of difficulty in swallowing these foods. Although bolus formation did not differ between mastication with and without hyposalivation, participants may have experienced dry conditions in the pharynx. Although particle size distribution and bolus lubrication are key determinants of swallowing initiation during mastication (Hutchings and Lillford 1988; Feldman et al. 1980), the perception of pharyngeal moisture should also be considered to determine readiness to swallow.

This study had several limitations. First, only a small number of healthy volunteers were recruited. Second, a fixed dose of atropine sulfate (1 mg) was used for all participants. Given that the effect of atropine might differ among subjects, the degree to which impaired salivation affects masticatory behavior should be clarified. Third, the study did not investigate swallowing behavior to evaluate how bolus transport and/or swallowing movements are affected by hyposalivation. Therefore, further studies are required to clarify how eating behaviors, including mastication and swallowing, are affected by hyposalivation.

## Author Contributions

**Chsato Aizawa:** investigation (lead), writing – review and editing (supporting). **Reiko Ita:** formal analysis (supporting), investigation (equal), writing – review and editing (supporting). **Yuto Ochiai:** formal analysis (supporting), investigation (equal), writing – review and editing (supporting). **Anna Sasa:** investigation (supporting), writing – review and editing (supporting). **Namon Phetnin:** investigation (supporting), writing – review and editing (supporting). **Yoko Kawana:** investigation (supporting), writing – review and editing (supporting). **Kazuhiro Ono:** conceptualization (equal), methodology (equal), validation (supporting), writing – review and editing (supporting). **Natsuka Takada:** conceptualization (equal), formal analysis (supporting), writing – review and editing (supporting). **Takanori Tsujimura:** formal analysis (equal), writing – original draft (supporting), writing – review and editing (equal). **Jin Magara:** formal analysis (equal), writing – original draft (supporting), writing – review and editing (equal). **Makoto Inoue:** conceptualization (lead), formal analysis (lead), funding acquisition (lead), methodology (lead), project administration (lead), resources (lead), software (lead), supervision (lead), validation (lead), writing – original draft (lead), writing – review and editing (lead).

## Funding

This work was supported in part by Japan Society for the Promotion of Science (22K10073) and research funding from Ichimasa Kamaboko Co. Ltd.

## Conflicts of Interest

The authors declare no conflicts of interest.

## Supporting information


**Table S1:** Correlation coefficient and *p* value for regression between masticatory cycle time and suprahyoid activity changes.

## Data Availability

All data supporting the findings of this study are available from the corresponding author upon request.
