# Supplementary material for: Effect of Hyposalivation on Fish Cake Mastication in Healthy Individuals
Source: Food Sci Nutr. 2026 Jan 19;14(1):e71378. doi: 10.1002/fsn3.71378 (PMC12816767; doi:10.1002/fsn3.71378)
Supplement: Supplementary file 1 — Table S1: Correlation coefficient and p value for regression between masticatory cycle time and suprahyoid activity changes. [file FSN3-14-e71378-s001.docx]

Supplemental table 1 Correlation coefficient and P value for regression between masticatory cycle time and suprahyoid activity changes.

|  | Pre |  |  | Post |  |
| --- | --- | --- | --- | --- | --- |
|  | CC | P value |  | CC | P value |
| Fish cake | 0.656 | 0.00167 |  | 0.727 | 0.000284* |
| Rolled omelet | 0.514 | 0.0206† |  | 0.783 | 0.0000441*,† |

*p < 0.05 vs Pre; †p < 0.05 vs Fish cake. CC, correlation coefficient; Post, after atropine administration; Pre, before atropine administration.
